# Early Attempts to Eradicate *Helicobacter pylori* after Endoscopic Resection of Gastric Neoplasm Significantly Improve Eradication Success Rates

**DOI:** 10.1371/journal.pone.0162258

**Published:** 2016-09-02

**Authors:** Cheal Wung Huh, Young Hoon Youn, Da Hyun Jung, Jae Jun Park, Jie-Hyun Kim, Hyojin Park

**Affiliations:** Department of Internal Medicine, Gangnam Severance Hospital, Yonsei University College of Medicine, Seoul, Korea; National Cancer Center, JAPAN

## Abstract

**Purpose:**

After endoscopic resection (ER) of gastric tumors, eradication of *Helicobacter pylori* (*H*. *pylori*) infection is advised to reduce metachronous recurrence. Optimal timing of such therapy (yet to be established) was investigated herein, examining early active and late scarring stages of post-ER iatrogenic ulcers.

**Materials and Methods:**

Analysis included 514 patients who received proton-pump inhibitor (PPI)-based triple therapy for *H*. *pylori* eradication after ER for gastric neoplasms between January 2008 and June 2015. Clinicopathologic characteristics, particularly the timing of triple therapy, were used to compare eradication rates, assigning patients to early- (≤2 weeks), intermediate- (2–8 weeks), and late-phase (≥8 weeks) treatment groups.

**Results:**

*H*. *pylori* eradication rates differed significantly by timing of triple therapy after ER (early, 90.0%; intermediate, 76.2%, late, 72.4%; *p* <.001). However, eradication success rates were not significantly affected by age, smoking, alcohol consumption, preexisting comorbidity, method of ER, size and location of iatrogenic ulcer, and duration of therapeutic regimen. Early initiation of *H*. *pylori* eradication was also identified as a significant independent predictor of eradication success in multivariate analysis (Odds ratio = 3.67, 95% CI 2.18–6.16; *p* <.001).

**Conclusion:**

In patients undergoing ER of gastric tumors, early post-ER attempts at eradication of *H*. *pylori* offer the best chance of eradication success.

## Introduction

Endoscopic resection (ER) is a widely accepted means of treating gastric adenomas and types of early gastric cancer (EGC), generally through endoscopic submucosal dissection (ESD) or endoscopic mucosal resection (EMR)[1,2]. Although the risk of developing a metachronous gastric neoplasm elsewhere in the stomach is relatively high after ER[3], many studies have shown that successful eradication of *Helicobacter pylori* (*H*. *pylori*) may reduce this risk[4–9]. As with peptic ulcer disease (PUD), eradication of *H*. *pylori* is thus an important therapeutic indication in this setting, dictated by post-ER status[6,7,9–11].

Researchers are actively seeking ways to improve eradication rate of *H*. *pylori*, given the gradually declining success rates due primarily to acquired antibiotic resistance[12–14]. Factors impacting eradication of *H*. *pylori* include smoking habits, alcohol consumption, age, body mass index, underlying disease, CYP2C19 genotype, existing PUD, patient compliance, regimen duration, and of course antibiotic resistance[15–17]. Results of a recent study further implicate the stage of PUD in eradication success[15]. However, few studies have addressed factors influencing *H*. *pylori* eradication rates in the aftermath of ER, and none have focused on the optimal timing of therapy to eradicate *H*. *pylori* post-ER.

Therefore, this study was conducted to evaluate whether differences in timing of eradication therapy after ER of gastric tumors (early active vs late scarring stages of iatrogenic ulcers) impacts success rates in eradicating *H*. *pylori*.

## Methods

### Patients

**Fig 1:**
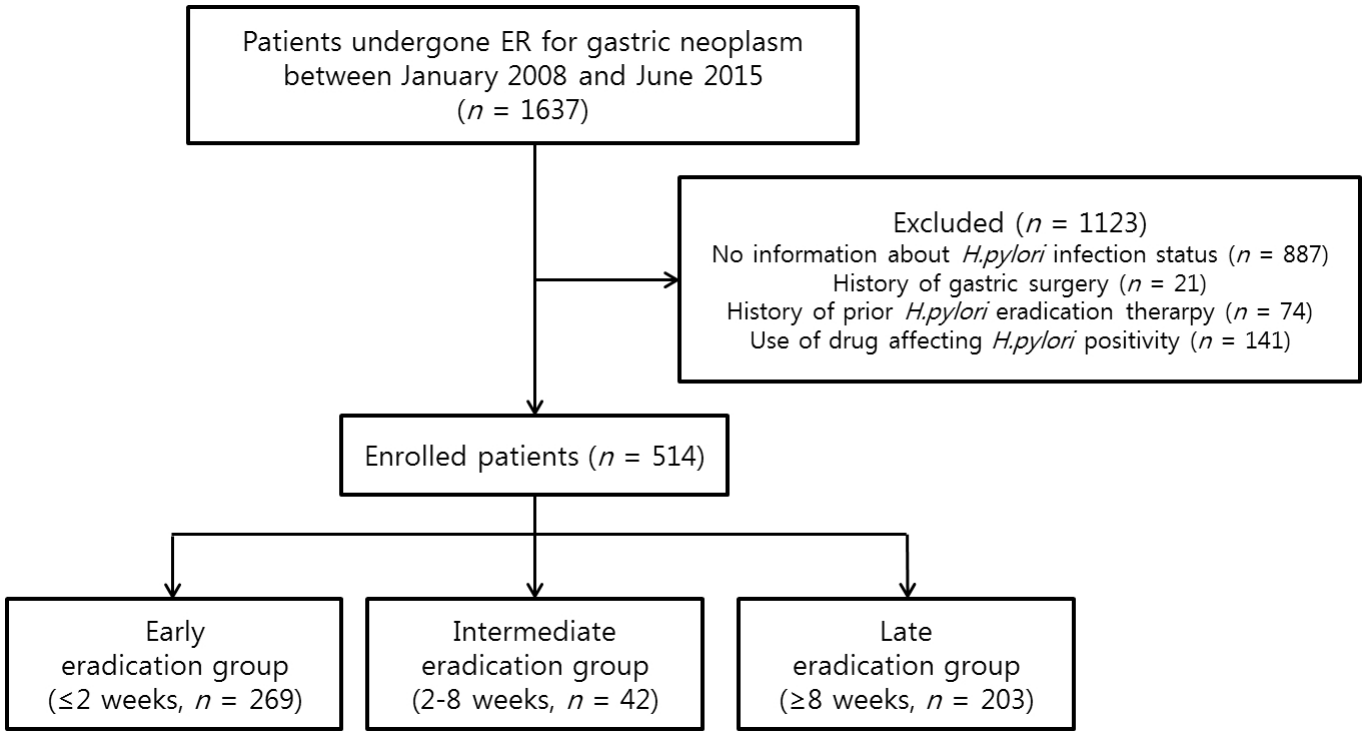
A flow chart of patient enrollment.

Overall, 1637 patients underwent ER of gastric neoplasms between January, 2008 and June, 2015 at Gangnam Severance Hospital. Among them, 514 post-ER recipients of proton-pump inhibitor (PPI)-based triple therapy (i.e., PPI [standard dosing], amoxicillin 1.0 gm, clarithromycin 500 mg; all taken bid) were selected for retrospective analysis. The patient selection and grouping flow diagram is shown in Fig 1. Histories of gastric surgery or prior *H*. *pylori* eradication therapy and use of drugs affecting *H*. *pylori* positivity (PPI, histamine-2 receptor blocker, or antibiotics) within the preceding 2 weeks were grounds for exclusion. The Institutional Review Board (IRB) of Gangnam Severance Hospital approved this study. We received a consent exemption from the IRB. Patients records and information was anonymized.

### Study design

In addition to timing and duration of *H*. *pylori* eradication, specific clinicopathologic factors, such as age, gender, smoking, alcohol consumption, preexisting comorbidity, method of ER (e.g., EMR or ESD), size and location of iatrogenic ulcer, endoscopic finding of background mucosa(e.g., mucosal atrophy or intestinal metaplasia) and histopathologic results, were collected from medical records. We defined the patients who smoke presently as “smoker” and the patient who drink more than 40g a day in men and more than 20g a day in women as as “alcoholics”. And, “preexisting comorbidity” included following disorders; diabetes, hypertension, heart disease, liver disease, kidney disease, pulmonary disease, etc. Atrophy and intestinal metaplasia were evaluated endoscopically at the time of ER. Atrophic gastric mucosa was defined by whitish to yellowish color change, visible submucosal vessels and absence of rugae. Intestinal metaplasia was characterized as metaplastic mucosa with fine or coarse plaques. The size of ulcers created via ER were extrapolated from the excised specimens. In the post-ER period, patients were grouped by timing (i.e., initiation) of therapy to eradicate *H*. *pylori* as follows: 1) early (≤ 2 weeks), 2) intermediate (2–8weeks) or 3) late (≥ 8weeks).

### Diagnosis and treatment of *H*. *pylori* infection

*H*. *pylori* infection was determined by histologic sections (hematoxylin & eosin and Wright-Giemsa stains), by rapid urease test (Asan Helicobacter Test; Asan Pharmaceutical Co Ltd, Seoul, Korea), and by serologic assay (IMMULITE 2000 *H*. *pylori* IgG; Diagnostic Products Corp [Siemens], Los Angeles, CA, USA). Infection was confirmed by either histologic means or rapid urease test positivity, or by positive serologic assay, in the absence of medication to eradicate *H*. *pylori*. Eradication of *H*. *pylori* was achieved through PPI-based triple therapy, consisting of PPI (standard dosing), amoxicillin (1.0 gm), and clarithromycin (500 mg), given twice daily for 7–14 days after ER. Initiation of therapy varied, depending on physician or patient preference, confirmation of infection (point in time), and follow-up scheduling. *H*. *pylori* eradication was established by urea breath test or rapid urease test at least 4 weeks after completion of triple therapy, and also at least 2 weeks after discontinuation of PPI or histamine-2 receptor blocker due to the possibility of false negative result.

### Statistical analysis

Between-group comparisons of clinicopathologic characteristics were conducted using chi-square or Fisher’s exact test, applying Student *t*-test for non-categorical variables. Factors impacting *H*. *pylori* eradication rates were subjected to logistic regression analysis. Statistical significance was set at *p*<0.05. All computations relied on standard software (SPSS v18.0 for Windows; SPSS Inc, Chicago, IL, USA).

## Results

### Demographic and clinicopathologic characteristics of subjects by eradication status

In this cohort, the mean rate of *H*. *pylori* eradication success was 81.9% (421/514). Clinicopathologic characteristics of the study population are shown in Table 1, stratified by eradication success (n = 421) and failure (n = 93). Age, smoking, alcohol consumption, underlying disease, mean size of iatrogenic ulcer, location of iatrogenic ulcer, background mucosal status, and tumor histology did not differ between groups. However, patient gender (male) and timing (early) of attempts to eradicate *H*. *pylori* showed statistically significant associations with *H*. *pylori* eradication rates (Table 1). Mean intervals from ER to initiation of triple therapy were 65.3±122.8 days in the eradication success group and 120.9±175.7 days in the eradication failure group. In addition, demographic characteristics among early treatment (≤2weeks), intermediate treatment (2–8weeks) and late treatement (≥8weeks) groups are shown in the table 2.

**Table 1:** Univariate analysis affecting the *Helicobacter pylori* eradication rate.

Variables	Success rate of eradication (%)	Eradication success (*N* = 421) (*n*, %)	Eradication failure (*N* = 93) (*n*, %)	*P*
Age (years, mean ± SD)	-	61.3 ± 10.7	59.9 ± 10.8	.230
Gender				.003
Male	85.4	293 (69.6)	50 (53.8)	
Female	74.9	128 (30.4)	43 (46.2)	
Smoking	82.4	61 (14.5)	13 (17.6)	.899
Drinking	82.3	188 (44.7)	40 (43.0)	.773
Preexisting comorbidity				.473
No	80.6	200 (47.5)	48 (51.6)	
Yes	83.1	221 (52.5)	45 (48.4)	
Method of ER				.110
EMR	87.0	100 (23.8)	15 (16.1)	
ESD	80.5	321 (76.2)	78 (83.9)	
Iatrogenic ulcer size (mm)	-	33.8 ± 15.5	34.8 ± 14.0	.565
Iatrogenic ulcer location				.988
Upper	80.5	35 (8.3)	8 (8.6)	
Middle	81.5	147 (34.9)	33 (35.5)	
Lower	82.3	239 (56.8)	52 (55.9)	
Endoscopic atrophy				.706
No	79.5	97 (23.0)	25 (26.9)	
Yes	82.0	324 (77.0)	68 (73.1)	
Endoscopic IM				.648
No	80.8	139 (33.0)	33 (35.5)	
Yes	82.5	282 (67.0)	60 (64.5)	
Tumor histology				.136
Cancer	78.8	197 (46.8)	53 (57.0)	
Dysplasia	83.7	169 (40.1)	33 (35.5)	
Other	88.7	55 (13.1)	7 (7.5)	
Duration of regimen				.207
7-d triple therapy	81.4	316 (75.0)	75 (80.6)	
10-d triple therapy	83.2	84 (20.0)	17 (18.3)	
14-d triple therapy	95.5	21 (5.0)	1 (1.1)	
Timing of HP eradication				<.001
Early (≤2weeks)	90.0	242 (57.5)	27 (29.0)	
Intermediate (2–8weeks)	76.2	32 (7.6)	10 (10.8)	
Late (≥8weeks)	72.4	147 (34.9)	56 (60.2)	

SD, standard deviation; ER, endoscopic resection; EMR, endoscopic mucosal resection; ESD, endoscopic submucosal dissection; IM, intestinal metaplasia; d, day; HP, *Helicobacter pylori*

**Table 2:** Comparisons of the demographic findings among early treatment, intermediate treatment, and late treatment groups.

Variables	Early treatment group (*N* = 269)	Intermediate treatment group (*N* = 42)	Late treatment group (*N* = 203)	*P*
Age (years, mean ± SD)	60.9 ± 11.3	60.3 ± 10.9	61.5 ± 9.8	.723
Gender				.171
Male	170 (63.2)	28 (66.7)	145 (71.4)	
Female	99 (36.8)	14 (33.3)	58 (28.6)	
Smoking	30 (11.2)	7 (16.7)	37 (18.2)	.087
Duration of regimen				.015
7-d triple therapy	205 (76.2)	26 (61.9)	160 (78.8)	
10-d triple therapy	47 (17.5)	14 (33.3)	40 (19.7)	
14-d triple therapy	17 (6.3)	2 (4.8)	3 (1.5)	
Success of HP eradication				<.001
Yes	242 (90.0)	32 (76.2)	147 (72.4)	
No	27 (10.0)	10 (23.8)	56 (27.6)	

SD, standard deviation; d, day; HP, *Helicobacter pylori*

Moreover, we have further analyzed with a data that excluded the patients in whom *H*.*pylori* infection was diagnosed by only serologic test (n = 146, 28.4%). This subgroup analysis also showed that the early treatment group (≤2 weeks, *n* = 146) also achieved a significantly higher eradication rate than did the intermediate (2–8 weeks, *n* = 27) or the late (≥8 weeks, *n* = 195) treatment group (early, 91.8%; intermediate, 74.1%; late, 72.3%; *p* <.001) (Table 3).

**Table 3:** Univariate analysis affecting the *Helicobacter pylori* eradication rate in subgroup in which diagnosis of infection was made by histology or rapid urease test.

Variables	Success rate of eradication (%)	Eradication success (*N* = 295) (*n*, %)	Eradication failure (*N* = 73) (*n*, %)	*P*
Age (years, mean ± SD)	-	60.9 ± 10.9	61.3 ± 10.5	.782
Gender				.061
Male	83.0	200 (67.8)	41 (56.2)	
Female	74.8	95 (32.2)	32 (43.8)	
Smoking	80.7	42 (14.2)	10 (13.7)	.987
Drinking	79.5	128 (43.4)	33 (45.2)	.960
Preexisting comorbidity				.776
No	79.5	140 (47.5)	36 (49.3)	
Yes	80.7	155 (52.5)	37 (50.7)	
Method of ER				.363
EMR	83.8	67 (22.7)	13 (17.8)	
ESD	79.2	228 (77.3)	60 (82.2)	
Iatrogenic ulcer size (mm)	-	33.2 ± 14.7	33.9 ± 14.0	.709
Iatrogenic ulcer location				.782
Upper	75.9	22 (7.5)	7 (9.6)	
Middle	79.5	97 (32.9)	25 (34.2)	
Lower	81.1	176 (59.6)	41 (56.2)	
Endoscopic atrophy				.965
No	81.2	69 (23.4)	16 (21.9)	
Yes	79.9	226 (76.6)	57 (78.1)	
Endoscopic IM				.581
No	78.6	99 (33.6)	27 (37.0)	
Yes	81.0	196 (66.4)	46 (63.0)	
Tumor histology				.056
Cancer	74.9	128 (43.4)	43 (58.9)	
Dysplasia	84.2	123 (41.7)	23 (31.5)	
Other	86.3	44 (14.9)	7 (9.6)	
Duration of regimen				.055
7-d triple therapy	78.0	216 (73.2)	61 (83.6)	
10-d triple therapy	83.6	61 (20.7)	12 (16.4)	
14-d triple therapy	100.0	18 (6.1)	0 (0.0)	
Timing of HP eradication				<.001
Early (≤2weeks)	91.8	134 (45.4)	12 (16.4)	
Intermediate (2–8weeks)	74.1	20 (6.8)	7 (7.6)	
Late (≥8weeks)	72.3	141 (47.8)	54 (74.0)	

SD, standard deviation; ER, endoscopic resection; EMR, endoscopic mucosal resection; ESD, endoscopic submucosal dissection; IM, intestinal metaplasia; d, day; HP, *Helicobacter pylori*

### *H*. *pylori* eradication rate relative to timing of triple therapy after ER

**Fig 2:**
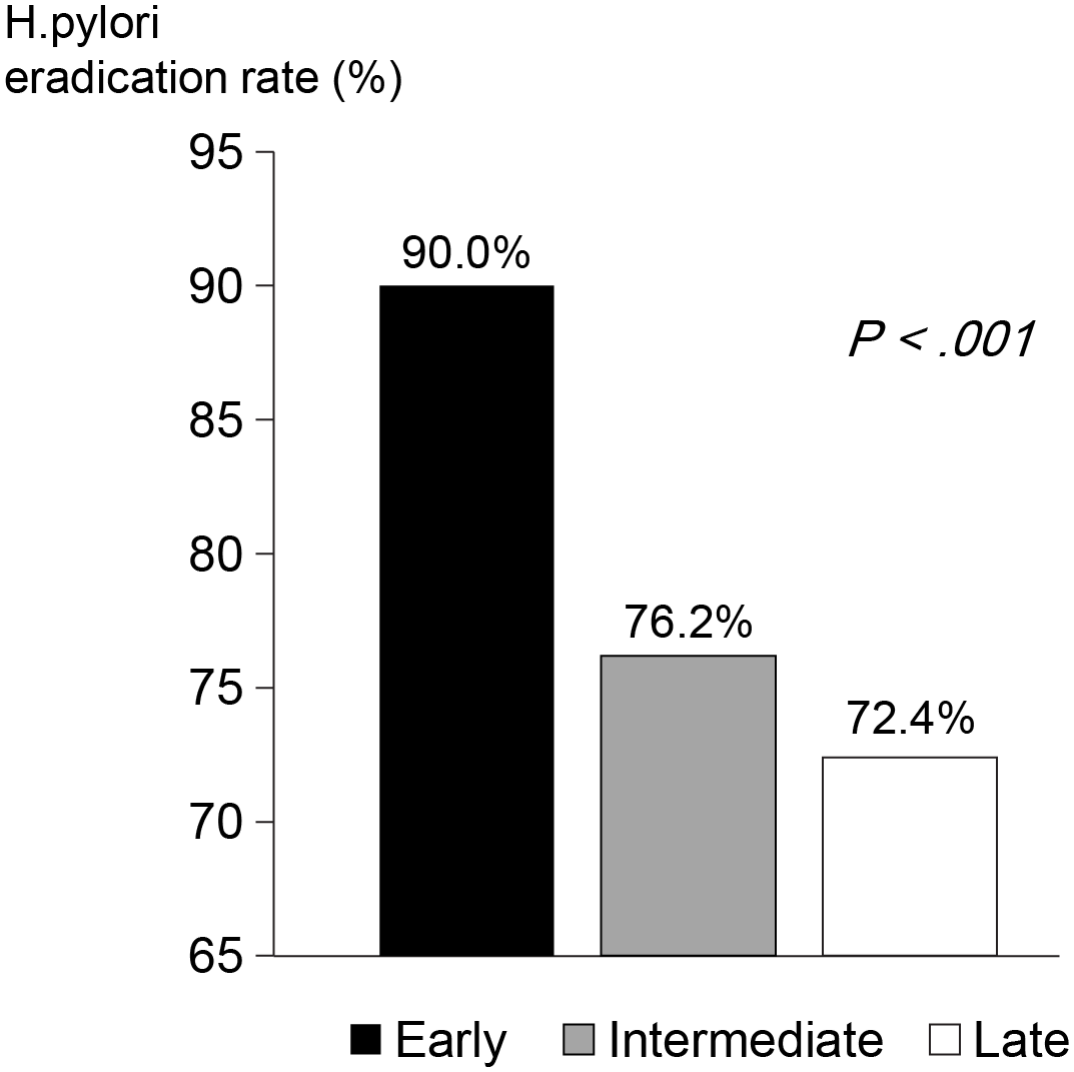
*H*. *pylori* eradication rate according to timing of therapeutic regimen after ER: a significantly higher eradication rate was achieved with treatment initiated early, compared with intermediate- or late-phase initiation (early, 90.0%; intermediate, 76.2%; late, 72.4%; *p* <.001).

The early treatment group (≤2 weeks, n = 269) achieved a significantly higher eradication rate than did the intermediate (2–8 weeks, n = 42) or the late (≥8 weeks, n = 203) treatment group (early, 90.0%; intermediate, 76.2%; late, 72.4%; *p* <.001) (Fig 2).

### Multivariate analysis of factors impacting *H*. *pylori* eradication rate

In multivariate analysis, assessing gender, duration of treatment(documented as significant factor in previous studies,[18] although they were not significant in our results), and timing of *H*. *pylori* eradication after ER, early initiation of *H*. *pylori* eradication and male gender were identified as significant independent predictors of eradication success (Odds ratio [OR] = 3.67, 95% CI 2.18–6.16; *p* <.001) (Table 4).

**Table 4:** Multivariate analysis affecting the *Helicobacter pylori* eradication rate.

Factors		Odds ratio (95% CI)	*P*
Gender	Female	1	
	Male	2.37 (1.46–3.84)	<.001
Duration of regimen	7-d triple therapy	1	
	10-d triple therapy	4.19 (0.53–32.77)	.171
	14-d triple therapy	3.22 (0.39–26.46)	.277
Timing to HP eradication	Late (≥8weeks)	1	
	Intermediate (2–8weeks)	3.14 (1.36–7.24)	.007
	Early (≤2weeks)	3.67 (2.18–6.16)	<.001

CI, Confidence interval; d, day; HP, *Helicobacter pylori*

## Discussion

To prevent metachronous lesions after ER of gastric tumors, especially in instances of EGC, eradication of *H*. *pylori* is an important therapeutic adjunct[4–9] that is incorporated in most treatment guidelines[19]. However, the optimal timing of eradicative therapy has yet to be addressed in this setting. Outcomes of the present study are the first to show that early initiation of such treatment after ER, in the active reparative phase of iatrogenic ulcers, is independently predictive of *H*. *pylori* eradication success (OR = 3.67, 95% CI 2.18–6.16; *p* <.001).

Success rates in eradicating *H*. *pylori* have gradually declined in many countries due to acquired microbial resistance to antibiotics. Recently, Shin *et al*. reported that *H*. *pylori* eradication rates with clarithromycin-containing triple therapy in Korea showed a decreasing trend over the past 10 years. The eradication rates of triple therapy ranged 84.9–87.5% from 2001 to 2007, and 80.0–81.4% from 2008 to 2010[20]. Although current guidelines[19,21,22] still recommend triple therapy (clarithromycin, amoxicillin, and a PPI) for 7–14 days as the standard eradication regimen, new strategies are urgently needed. In this study, the overall eradication rate of triple therapy was somewhat high because early eradication group achieved a significantly high eradication rate (overall, 81.9%; early, 90.0%; intermediate, 76.2%; late, 72.4%).

Many studies have found that post-ER iatrogenic ulcers tend to heal within 8 weeks[23–25], supporting Sakita’s three-stage reparative classification as follows [26]: 1) active-to-healing stage (≤ 2 weeks), 2) healing-to-scarring stage (2–8 weeks), and 3) scarring stage (≥ 8weeks). Our discovery that early initiation of triple therapy after ER is independently predictive of eradication success may then be linked with ongoing active gastric repair at sites of ER. Previous investigations have also reported that *H*. *pylori* eradication rates are higher in patients with PUD than in patients with non-ulcerative dyspepsia[16,27,28]. Moreover, Seo *et al* reported that active ulcers were likewise found to be independent predictors of successful eradication and concluded that early *H*.*pylori* eradication should be considered in patients with peptic ulcer bleeding for higher eradication rate[15]. Unfortunately, the mechanisms responsible for differing eradication rates as ulcers heal remain unclear.

The disparate levels of inflammatory immune reactivity inherent in active and scarred gastric ulcers or defects may offer one explanation. In this regard, a significantly higher eradication rate has been observed by Malfertheiner *et al*, correlating with intense (vs mild) neutrophilic infiltration of gastric antrum[29]. It may well be that therapeutic efficacy is improved in the absence of gastric mucus and epithelial layers, thus encouraging penetration of charged antibiotics from stomach lumen and facilitating systemic drug delivery through altered epithelial and vascular permeability[27]. Hence, inflammatory reactions quite conceivably may render *H*. *pylori* infections more susceptible to antibiotic therapy, conferring higher eradication rates in the context of active repair.

Our analysis also revealed a significant association between male gender and *H*. *pylori* eradication success, although this finding appears controversial. Moayyedi *et al* have reported similar results, citing gender-related differences in gastric physiology[30], and recently, Lee *et al* provided further confirmation[31]. However, no such relationship was evident in another study[32], casting doubt on the impact of gender in eradication of *H*. *pylori*.

There was no guideline regarding the optimal timing of *H*. *pylori* eradication in post-ER setting. As noted by Cheon *et al*., early therapy to eradicate *H*. *pylori* may additionally serve to speed the healing of post-ER iatrogenic ulcers[33]. However, because patients may experience nausea, dyspepsia or abdominal pain immediately after ER[34], some physicians prefer to delay *H*. *pylori* eradication for this reason. We nevertheless maintain that early initiation of therapy after ER is a sound rationale, capable of enhancing eradication success. However, early eradication therapy should be reconsidered in patients with acute symptoms after ER (e.g., nausea, dyspepsia or abdominal pain) for it may affect not only the patient’s quality of life but also completion rate of eradication therapy.

Our study has several acknowledged limitations. First, the analysis was retrospective and non-randomized, causing unavoidable potential for selection bias. And for the same reason, we could not evaluate the side effects and completion rates of eradication therapy in each groups. In addition, the duration of regimen showed differences among three groups (early treatment, intermediate treatment, late treatment groups). Although there was no significant difference in eradication rate of *H*.*pylori* according to the duration of regimen (*p* = 0.207), it is also a limitation of this study. Finally, assays of bacterial antibiotic resistance were not feasible. Therefore, further randomized prospective study is needed to validate our result. Nevertheless, our result yields important clues in clinical practice as to when *H*. *pylori* eradication should be initiated in patients who underwent ER for gastric neoplasm.

In conclusion, early treatment to eradicate *H*. *pylori*, initiated ≤2 weeks after ER of gastric tumors, emerged in this study as an independent predictor of eradication success and is thus encouraged as an important therapeutic adjunct, although further prospective study is needed to validate our result.
